# Integrating uniform design and response surface methodology to optimize thiacloprid suspension

**DOI:** 10.1038/srep46018

**Published:** 2017-04-06

**Authors:** Bei-xing Li, Wei-chang Wang, Xian-peng Zhang, Da-xia Zhang, Wei Mu, Feng Liu

**Affiliations:** 1 Shandong Provincial Key Laboratory for Biology of Vegetable Diseases and Insect Pests, College of Plant Protection, Shandong Agricultural University, Tai’an, Shandong 271018, P. R. China; 2 Research Center of Pesticide Environmental Toxicology, Shandong Agricultural University, Tai’an, Shandong 271018, China; 3 Key Laboratory of Pesticide Toxicology & Application Technique, Shandong Agricultural University, Tai’an, Shandong 271018, P. R. China

## Abstract

A model 25% suspension concentrate (SC) of thiacloprid was adopted to evaluate an integrative approach of uniform design and response surface methodology. Tersperse2700, PE1601, xanthan gum and veegum were the four experimental factors, and the aqueous separation ratio and viscosity were the two dependent variables. Linear and quadratic polynomial models of stepwise regression and partial least squares were adopted to test the fit of the experimental data. Verification tests revealed satisfactory agreement between the experimental and predicted data. The measured values for the aqueous separation ratio and viscosity were 3.45% and 278.8 mPa·s, respectively, and the relative errors of the predicted values were 9.57% and 2.65%, respectively (prepared under the proposed conditions). Comprehensive benefits could also be obtained by appropriately adjusting the amount of certain adjuvants based on practical requirements. Integrating uniform design and response surface methodology is an effective strategy for optimizing SC formulas.

Suspension systems are commonly used for various products including food, pharmaceuticals, paint and nanomaterials^1^,^2^,^3^. Their application in pesticides has also attracted tremendous attention because of the great interest in creating a cleaner environment^4^. A suspension concentrate (SC) has immense advantages over an emulsion concentrate. First, the primary medium in an SC formula is water instead of an organic solvent, which tends to enhance its social and economic benefits^5^. In addition, the small particle size of SC products can significantly improve the dispersibility, spreadability and biological activity of the active ingredient^6^. Furthermore, an SC has higher dilution stability in regard to the tank mixture. However, SCs are thermodynamically unstable systems, which always produce aqueous separation, flocculation or agglomeration^4^. All of these undesirable phenomena can decrease the commercial performance of SC products. Dispersing agents and anti-settling agents are often added to promote physical stability. However, optimization procedures have always been conducted based on single-factor experiments. A typical SC product consists of solid technical material (the active ingredient), wetting and dispersing agents, and anti-settling agents. Thus, there may be various interactions between the components, and these interactions have frequently been neglected in previous publications^7^,^8^.

Response surface methodology (RSM) is the most effective experimental design to achieve such optimization by improving experimental procedures^9^,^10^,^11^. It is especially appropriate for modelling and analysing multi-factor experiments to clarify how the single and interaction effects influence the responses based on a series of statistical techniques^12^,^13^. Currently, RSM has been widely applied in food chemistry, pharmaceuticals, material science, chemical engineering and other fields^14^. We have also demonstrated its favourable adaptability in pesticide formulations^15^,^16^. However, the number of proposed conditions in RSM is excessive. Central composite design and Box-Behnken design are the two traditional experimental designs most used in RSM^17^,^18^,^19^. Taking central composite design as an example, the number of conditions for five-factor experiments reaches fifty, and the number is still as high as thirty for a three-factor experiment.

To overcome this disadvantage, the combination of uniform design and RSM may be effective. Uniform design was first proposed by Fang to reduce the number of conditions for missile designs in 1978^20^. This integrated approach has been successfully applied in chemical engineering^21^,^22^, bio-engineering^23^,^24^ and civil engineering^25^. Uniform design considers only a uniform distribution of experimental points with high representativeness in the experimental region^26^. Specifically, the number of proposed experiments is determined only by the factor levels^20^. Therefore, uniform design has tremendous advantages in terms of the number of experimental conditions compared to RSM, triangular diagram, orthogonal design and other partial test methods.

Thiacloprid, a second neonicotinoid insecticide, was first patented by Bayer Crop Science and then launched with the brand name Calypso^27^,^28^. It shows high activity against sucking insects such as aphids, jassids and whiteflies^29^,^30^. We successfully optimized a thiacloprid 25% SC using RSM in previous studies^16^, but the optimization capability of a uniform design with fewer experiments remained unknown. We adopted an integrative approach of uniform design and RSM in this work to determine its merits in optimizing the thiacloprid SC formula. This optimization strategy is expected to be widely used in various engineering and technological fields.

## Results and Discussion

### Wetting, suspending and anti-settling properties of selected agents

**Figure 1:**
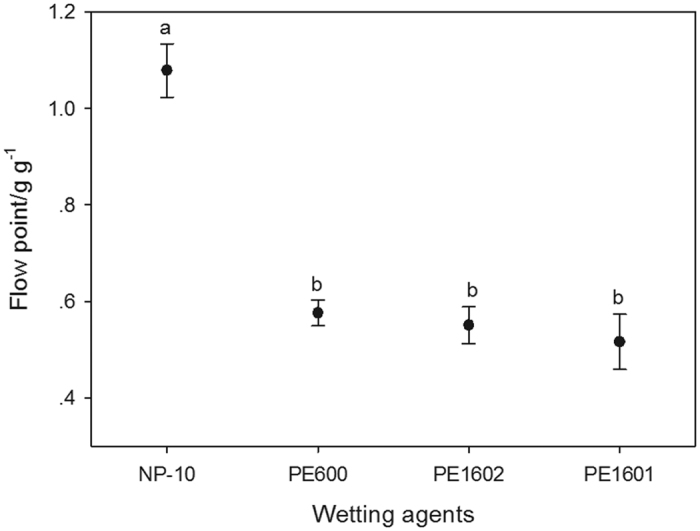
Flow points of different wetting agents. Data are displayed as the mean ± SD. Data with different lower case letters are significantly different at the *p* < 0.05 level according to Tukey’s test.

The flow points of different wetting agents were measured to assess their wettability and dispersibility, and the results are depicted in Fig. 1. It was apparent that NP-10 exhibited the worst wetting performance of the thiacloprid technical material among the four surfactants tested. PE600, PE1602 and PE 1601 showed no significant difference in terms of flow points, though PE1601 was observed to possess the most favourable wettability. As shown in Figure S1 (Supplementary Information), a PE1601 molecule has an analogous structure to the segmented copolymer EO-PO-EO (Ethylene oxide - Propylene epoxide - Ethylene oxide), which enables it to act as a wetting agent^16^. Although the hydrophilic EO group provides favourable wettability, it can cause large quantities of foam. Thus, SAG 630, an organosilicon defoamer, was added to lower the surface tension. Moreover, PE1601 offers the definite function of steric stabilization^31^. However, this weak stabilization cannot support a stable suspension. Combining non-ionic surfactants and anionic surfactants is a crucial strategy for promoting the physical stability of pesticide suspensions. Polycarboxylate surfactants are the most efficient category, among which Tersperse2700 has attracted intensive attention. As shown in Figure S2 (Supplementary Information), there are high levels of carboxyl in the molecular structure of Tersperse2700, which produce an effective charge repulsion to inhibit aggregation between suspended particles^32^. Moreover, favourable steric hindrance created out by the macromolecular structure of Tersperse2700 should not be neglected. In the current study, PE1601 and Tersperse2700 were used simultaneously to provide wetting or dispersing functions, and they were adopted as the independent variables herein. Xanthan gum is an anionic polysaccharide with the versatile ability to form hydrogels through hydrogen bonding or inter-molecular electrostatic interactions. A xanthan gum solution was reported to possess shear-thinning properties^33^. Providing the SC with high viscosity generates a significant advantage in terms of reducing the settling action according to Stokes’ law. More importantly, the SC became very thin upon applying stress or strain, and therefore this type of liquid preparation was optimal for dilution. Magnesium aluminium silicate was also a component capable of promoting the thixotropy of suspended systems. Moreover, our previous studies demonstrated that the physical stability of SCs can be significantly ameliorated by combining magnesium aluminium silicate and xanthan gum, mainly due to a synergistic effect^16^; thus, both were used to enhance the anti-settling properties of thiacloprid SC.

### Quality control indexes of thiacloprid SC

The corresponding responses of the uniform design are shown in Table 1. Fortunately, all nine samples exhibited favourable fluidity. However, the aqueous separation ratios and viscosities of experimental runs differed significantly. Specifically, the aqueous separation ratios ranged from 1.74% to 30.23%, while the viscosities of the tested samples ranged from 267.8 to 364.9 mPa·s. Measurements of sample suspensibility were carried out both before and after the accelerated storage procedure. All samples showed favourable suspensibility before thermal storage, with values ranging from 92.89% to 97.62%. After storage, the suspensibility values of the samples were also higher than 91%, although slight decreases were observed. As Stokes’ law states that small particles settle much more slowly, we speculated that the high suspensibility values were mainly attributable to the small particle size and uniform size distribution (Table S1, Supplementary Information). Although a small degree of Ostwald ripening was observed for all samples, the median diameter of most samples was approximately 2 μm even after accelerated storage, which provided the fundamental prerequisites for favourable suspensibility. Fortunately, this level of suspensibility is generally agreed to ensure good performance for most types of SC^34^. Therefore, no further optimization with regard to suspensibility was required in the current study. Because no apparent rules were found that could predict the aqueous separation ratios and viscosities of the samples, the data of the two independent variables were analysed. We expected to obtain a preparation with a low aqueous separation ratio and relatively low viscosity through a thorough analysis of the dependent variables.

### Optimization of the aqueous separation ratio

A regression analysis was performed to fit the aqueous separation ratios using DPS software (version 7.05). Stepwise regression and partial least squares were reported to best fit the results obtained from the uniform design^35^,^36^. In the current study, linear and quadratic polynomial models of stepwise regression and partial least squares, respectively, were therefore adopted to test the fitness. As shown in Table 2, all four models exhibited favourable fitness with coefficients of determination (R^2^) greater than 0.94. Among the models, the quadratic polynomial model of stepwise regression not only exhibited the greatest R^2^, but also indicated a significant model with F_(4,4)_ = 51.57 (F_0.05(4,4)_ = 6.39). The F-value is a statistic derived from the Fischer test that indicates whether the data deviate significantly from the mean. When the F-values of the experimental data are greater than critical F-values (at a certain *p* level; the most frequently used level is *p* = 0.05), we judge that the selected models are eligible. The *p*-value is a more direct index that implies the significance of the data. Specifically, a *p*-value smaller than 0.05 indicates a significant model. The *p*-value of the quadratic polynomial model of stepwise regression was only 0.0011, which showed adequate significance. Thus, the regression equation of the best model was as follows:


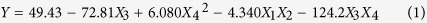


where *Y* is the aqueous separation ratio and *X*_1_, *X*_2_, *X*_3_ and *X*_4_ represent Tersperse2700, PE1601, xanthan gum and veegum, respectively.

**Figure 2:**
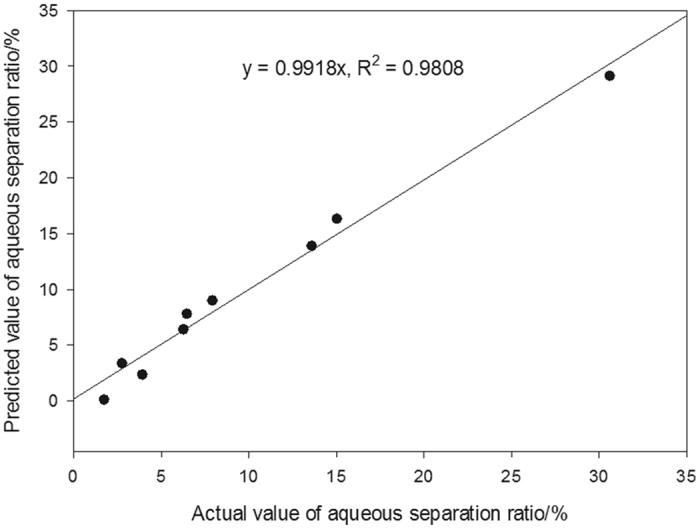
Predicted and actual values of the aqueous separation ratio.

This result is further confirmed by Fig. 2, in which the predicted and actual values are regressed. The predicted values were generated after calculating the amounts of *X*_1_, *X*_2_, *X*_3_ and *X*_4_ using Equation 1. As depicted in Fig. 2, the empirical equation fits the experimental data well. We sought to decrease the aqueous separation ratio as much as possible to obtain adequate physical stability for the SC. It was clear that the aqueous separation ratio decreased as the amounts of *X*_1_, *X*_2_ or *X*_3_ increased, regardless of the other independent variables. In terms of *X*_4_, the prediction of the aqueous separation ratio became more complicated. When 0.22% *X*_3_ was added (highest level) and *X*_1_ and *X*_2_ were at the optimal levels, the aqueous separation ratio decreased with increasing amount of *X*_4_. When 0.14% *X*_3_ was added (lowest level) and *X*_1_ and *X*_2_ were at the optimal levels, the equation could be simplified to “*Y* = 6.080(*X*_4_ −1.430)^2^ −5.746”. Clearly, the addition of more than 1.43% *X*_4_ would increase the aqueous separation ratio. Further research should explore whether there are more visual and/or convenient methods for obtaining favourable conditions.

**Figure 3:**
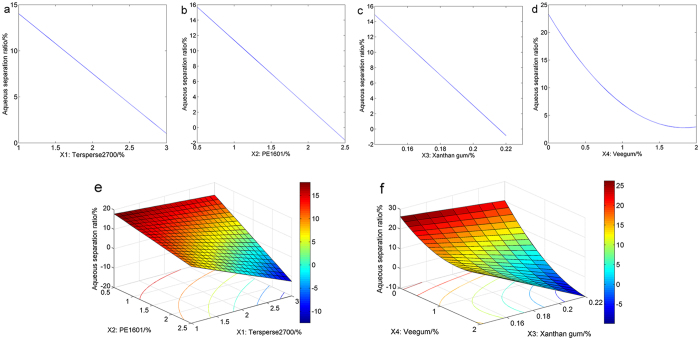
Response surface plots for the aqueous separation ratio.

Response surface plots are highly beneficial for clarifying interactions between dependent variables, and they also generate optimal responses with balanced conditions. As elaborated above, favourable conditions were obtained for the highest levels. However, such extreme conditions are always difficult to generate or control, which in turn may increase the potential for unsuccessful prediction^37^. Therefore, three variables were maintained at median levels while the other variable ranged from the lowest to the highest level to investigate the respective influence of the four factors. Significant decreases in the aqueous separation ratios were observed with increasing amounts of *X*_1_, *X*_2_, *X*_3_ and *X*_4_ (Fig. 3a,b,c and d). However, when the amount of veegum reached 1.5%, the aqueous separation ratios only changed slightly, as depicted in Fig. 3d. Subsequently, with the aqueous separation ratio as the response, response surfaces illustrating the interactions between *X*_1_ and *X*_2_ (Fig. 3e) and between *X*_3_ and *X*_4_ (Fig. 3f) were plotted when the other two variables were maintained at median levels. As shown in Fig. 3e, the aqueous separation ratio dropped dramatically with variations in the amounts of *X*_1_ and *X*_2_, especially when they were maintained at higher levels. When *X*_3_ was maintained at a low level, the aqueous separation ratio decreased gradually as the amount of *X*_4_ decreased (Fig. 3f). However, when a high amount of *X*_3_ was added, the aqueous separation ratio dropped abruptly with the amount of *X*_4_. This result is significantly different from our previous finding^16^, in which response plots were illustrated under optimal conditions of independent variables. We suppose that maintaining moderate experimental conditions may be a more reasonable strategy for obtaining better predictive accuracy. Therefore, further research is still required.

### Optimization of viscosity

Linear and quadratic polynomial models of stepwise regression and partial least squares, respectively, were used to test the fit to the data. The regression results of viscosity are shown in Table S2 (Supplementary Information). All four models showed acceptable fits with R^2^ > 0.96. Among them, the quadratic polynomial model of stepwise regression best fit the experimental data, yielding R^2^ = 1.000. The proposed model was a significant model with F_(7,1)_ = 7.143 × 10^4^, and the *p*-value was 0.0029. The regression equation of the best model is as follows:


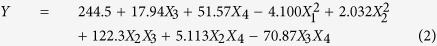


The predicted viscosity values were calculated using Equation 2 and then regressed with actual values, as depicted in Figure S3 (Supplementary Information). The empirical equation fit the experimental data well, which was consistent with Fischer’s test. To ensure adequate pourability of the preparations, the SC should possess low or at least moderate viscosity. Equation 2 indicates that the viscosity decreased with increasing amount of *X*_1_ and increased with *X*_2_, regardless of the other independent variables. However, it was difficult to determine how to adjust the amounts of *X*_3_ and *X*_4_ to yield the lowest viscosity by observing the proposed regression equation. Therefore, the “fmincon function” in MATLAB was used to generate the optimal conditions for the lowest viscosity. The optimal conditions were determined to be adding *X*_1_, *X*_2_, *X*_3_ and *X*_4_ at mass fractions of 3%, 0.5%, 0.14% and 0, respectively. The minimal viscosity was estimated to be 241.2 mPa s.

**Figure 4:**
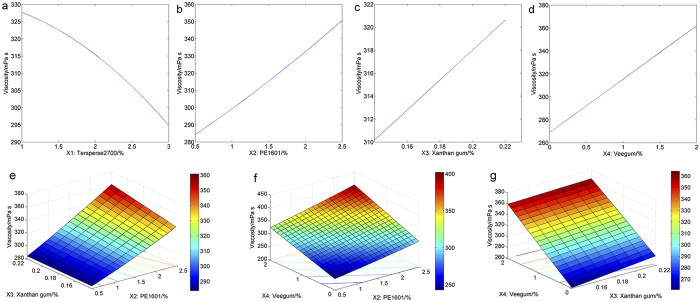
Response surface plots for viscosity.

To visualize these relationships further, response surfaces were also plotted to reveal the influence of the tested factors on viscosity. As stated above, three variables were maintained at median levels while the other variable ranged from the lowest level to the highest level to investigate the respective influence of the four factors. As illustrated in Fig. 4a, the viscosity decreased dramatically with increasing amounts of *X*_1_. However, significant increases in viscosity were observed with an increase in *X*_2_, *X*_3_ or *X*_4_ (Fig. 4b,c and d). The interactions between *X*_2_ and *X*_3_, *X*_2_ and *X*_4_, and *X*_3_ and *X*_4_ were significant and are displayed in Fig. 4. The viscosity increased significantly with the amount of *X*_2_ regardless of the amount of *X*_3_ added, as shown in Fig. 4e. When *X*_2_ was maintained at the lowest level, the viscosity varied little with variations in the amount of *X*_3_. However, when *X*_2_ was added at the highest level, an increase of approximately 30 mPa s in viscosity was observed with increasing amounts of *X*_3_ (Fig. 4e). As depicted in Fig. 4f, viscosity increased significantly with the levels of *X*_2_ and *X*_4_ when *X*_1_ and *X*_3_ were maintained at median levels. In terms of the interaction between *X*_3_ and *X*_4_, viscosity varied slightly with the amount of *X*_3_ but increased dramatically with *X*_4_ regardless of the *X*_3_ level (Fig. 4g).

### Optimization of multiple-responses

**Figure 5:**
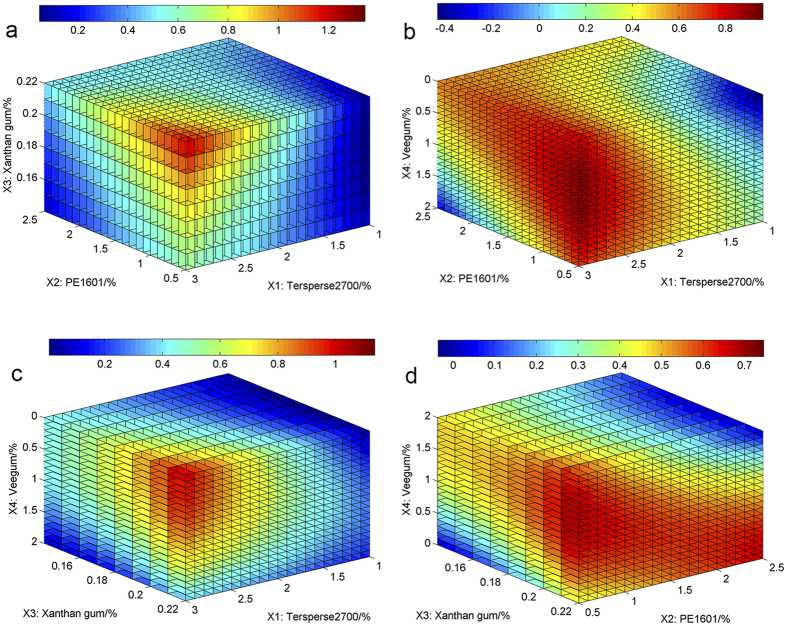
Four-dimensional response surfaces that reveal the influences of *X*_1_*X*_2_*X*_3_, *X*_1_*X*_2_*X*_4_, *X*_1_*X*_3_*X*_4_, and *X*_2_*X*_3_*X*_4_. The colour bar represents the total desirability.

In the current study, the aqueous separation ratio and viscosity are two independent responses. However, the optimal responses were achieved under different conditions. Thus, a desirability function was employed to optimize the parameters through a compromise among the conditions for the two output responses. The aqueous separation ratio and viscosity seem equally important in influencing the performance of an SC preparation. Therefore, the relative weights of the aqueous separation ratio and viscosity were both set as 1. The resulting output individual and total desirability are listed in Table S3 (Supplementary Information). However, no obvious rule could be observed regarding the total desirability. Our main objective is to maximize the total desirability, and thus the total desirability data were fitted using the four models mentioned above. As shown in Table S4 (Supplementary Information), the quadratic polynomial model of partial least squares fits the total desirability data best (with the highest R^2^ and lower residuals). The regression equation of the best model is as follows:


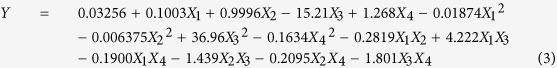


where *Y* is the total desirability. The “fmincon” function in MATLAB was used to generate the optimal conditions for the lowest “−*Y*” i.e., the highest “*Y*”. The optimal conditions were determined to be the addition of *X*_1_, *X*_2_, *X*_3_ and *X*_4_ at mass fractions of 3%, 0.5%, 0.22% and 0.60%, respectively. The maximal total desirability was estimated at 1.37. Generally, an SC product with an aqueous separation ratio lower than 5% and a viscosity lower than 300 mPa s ensured adequate favourable fluidity, dispersibility and physical stability^5^,^6^. The total desirability derived from these conditions was calculated to be 0.77. Thus we deduced that total desirability higher than 0.80 was adequate, even if the most critical performance was required. However, the levels of variability that yielded the maximum were achieved with the highest levels of *X*_1_ and *X*_3_. In this case, emphasis on generating the largest total desirability by maintaining *X*_1_ and *X*_3_ at their highest levels was a wasteful effort given the increasing potential for unsuccessful prediction under such extreme conditions^37^. Subsequently, response surfaces revealing the influences of *X*_1_*X*_2_*X*_3_, *X*_1_*X*_2_*X*_4_, *X*_1_*X*_3_*X*_4_, and *X*_2_*X*_3_*X*_4_ were plotted by maintaining other variables at median levels. As illustrated in Fig. 5a, the total desirability increased dramatically with increasing levels of *X*_1_ and *X*_3_. When the *X*_1_, *X*_2_ and *X*_3_ levels ranged from 2.50 to 3.00%, 0.50 to 1.10% and 0.19 to 0.22%, respectively, the total desirability was always higher than 0.80 (Fig. 5a). As shown in Fig. 5b, favourable total desirability was only obtained when the *X*_1_, *X*_2_ and *X*_4_ amounts ranged from 2.80 to 3.00%, 0.20 to 0.90% and 0.20 to 1.60%, respectively (crimson areas). When the *X*_1_, *X*_3_ and *X*_4_ amounts ranged from 2.60 to 3.00%, 0.20 to 0.22% and 0 to 1.10%, respectively, the total desirability was always higher than 0.80 (Fig. 5c). However, favourable total desirability was rarely obtained when *X*_1_ was maintained at median levels (Fig. 5d), which demonstrated the importance of adding high levels of *X*_1_. As Fig. 5d illustrates, maintaining *X*_2_, *X*_3_ and *X*_4_ amounts between 0.50 and 0.70%, 0.21 and 0.22%, and 0.70 to 1.70%, respectively, was reasonably appropriate for yielding the highest total desirability. Many solutions were adequate when only the preparation technology was considered. However, Tersperse2700 (*X*_1_) is far more expensive than other adjuvants. Therefore, we desired to use as little Tersperse2700 as possible due to financial constraints. Maintaining *X*_1_ = 2.50%, the amounts of *X*_2_, *X*_3_ and *X*_4_ ranging from 0.50 to 0.70%, 0.21 to 0.22% and 0.70 to 1.10%, respectively, were preferred (Fig. 5a,b,c and d). Two verification samples were then prepared and tested, as shown in Table 3. The predicted values were calculated according to equations (1) and (2). The measured values for the aqueous separation ratio and viscosity were 3.45% ± 0.16% and 278.8 ± 2.5 mPa s (mean ± SD). The relative errors of the predicted values above were 9.57% and 2.65%. Unfortunately, the relative errors of the predicted values for sample No. 1 were 64.50% and 7.49%. We found that even integrating the uniform design and response surface methodology can lead to prediction failure. Although the uniform design showed remarkable advantages in multi-factorial experiments, the instability of the predictions also increased relative to the central composite design^16^. Thus we suggested that (1) more verification points could be selected among the proposed levels of independent variables and (2) uniform design tables with larger degrees of freedom could be adopted if failure occurs when more variables are present.

## Conclusion

In this study, a uniform design table, U_9_(9^4^), was integrated with the response surface methodology to clarify how the tested factors influenced the dependent variables. This strategy exhibited adequate performance in optimizing the formula of a thiacloprid SC. Comprehensive benefits could also be obtained by appropriately adjusting the amount of certain adjuvants based on practical requirements.

## Materials and Methods

The thiacloprid technical material (purity >95%) was purchased from Shandong Sino-Agri United Biotechnology Co., Ltd. (Shandong, China). Pesticide emulsifier (PE) 1601, PE1602, PE600 and NP-10 (nonylphenol polyoxyethylene ether) were all purchased from Xingtai Lanxing Auxiliary Factory, Hebei, China. Tersperse2700 (polycarboxylate, M_w_ = 7808) is a high-efficiency dispersant provided by Huntsman (Salt Lake City, USA). Xanthan gum was purchased from Deosen Biochemical Ltd., Shandong, China. Veegum (magnesium aluminium silicate) was provided by Sinoma Mineral Materials Company, Jiangsu, China.

### Screening of wetting agents

The flow point was measured to assess the wetting ability of the wetting agents^38^. The standard procedure is described as follows: first, the technical material was smashed using a jet mill to obtain an average diameter of approximately 5 μm. Then, an aqueous solution containing 5% (w/w) tested surfactant was added dropwise to wet 15 g of technical material (*m*_1_). Next, we obtained a pasty mixture after stirring with a glass rod. When the mixture could fall freely, the added weight of this solution (*m*_2_) was recorded. The flow point was defined as the ratio of *m*_2_ to *m*_1_. In general, wetting agents with lower flow points were considered to possess favourable wettability.

### SC preparation

We adopted the wet grinding method to generate the thiacloprid SC in the current study. First, 52.63 g of thiacloprid technical material; specified weights of wetting agents (with favourable wettability), suspending agents and anti-settling agents; 4 g of glycerol and 1 g of defoamer were accurately weighed. Then, distilled water was added until the mixture totalled 200 g. Next, the coarse mixture was transferred to a stainless steel cup with isopyknic zirconium oxide beads. Finally, the mixture was ground at 1700 r min^−1^ for 1 h to yield a homogeneous 25% SC of thiacloprid. Cooling water was used throughout the process to maintain relatively stable grinding conditions.

### Uniform design

Experiments were proposed according to a uniform design table, namely, U_n_(q^s^), where U represents the uniform design, n indicates the number of proposed experiments, q describes the level, and s is the number of table columns. The U_9_(9^4^) table (centred L_2_-discrepancy was 0.0478; wrap-around L_2_-discrepancy was 0.2356) was used in this study to clarify how the factors influenced the dependent variables. The factors and levels of the uniform design are shown in Table S5 (Supplementary Information). Four adjuvants (Tersperse2700, PE1601, xanthan gum and veegum) were set as the experimental factors, and there were nine levels for each factor.

### Optimization of multiple responses

A desirability function was employed to optimize the parameters through a compromise among the conditions for the two output responses. Smaller-The-Best, a response type proposed by Derringer and Suich, was adopted^39^. The individual desirability function is defined as d = (Y_max_ − Y)/(Y_max_ − Y_min_), where Y_max_ and Y_min_ indicate the maximum (30.63% for aqueous separation ratio and 364.9 mPa·s for viscosity) and minimum (1.74% for aqueous separation ratio and 267.8·mPa s for viscosity), respectively^40^. The total desirability was computed as a weighted geometric mean of the individual desirability functions^41^: 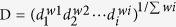, where d_i_ is the individual desirability of the i-th response, wi is the relative weight, and D is the total desirability.

### Measuring quality control indexes

An accelerated storage procedure was conducted to simulate the normal long-term physical stability of the SC, in accordance with our previous publications^16^. All samples were placed in an oven for 14 days at a constant temperature of 54 ± 2 °C. The aqueous separation ratios of all samples were then measured to assess the thermal physical stability. The aqueous separation ratio was defined as the ratio of *m*_*up*_/*m*_*to*_, where *m*_*up*_ and *m*_*to*_ are the weight of the separated aqueous phase after thermal storage and the total weight of the sample, respectively. To enhance the experimental precision, all measurements were conducted in tripartite, and the data were presented as the mean ± SD. Samples with lower aqueous separation ratios indicate better physical stability. A laser particle size analyser (Zhuhai OMEC instrument Co., Ltd., Guangdong, China) was used to evaluate the size distribution of the thiacloprid SC. Measurements were repeated in quadruplicate, and D_10_ (particle size of 10% cumulative distribution), D_90_ and the median diameter (D_50_) were selected as the main parameters. Viscosity was determined using a rheometer (Brookfield, Massachusetts, USA) according to a previously reported method. Measurements of the suspensibility of samples were performed using the method recommended in MT161 Suspensibility of Aqueous Suspension Concentrates (drafted by the Collaborative International Pesticides Analytical Council).

### Statistical Analysis

All of the data were statistically analysed using DPS software (version 7.05), and three-dimensional and four-dimensional diagrams were drawn by MATLAB R2014a.

## Additional Information

**How to cite this article**: Li, B.-x. *et al*. Integrating uniform design and response surface methodology to optimize thiacloprid suspension. *Sci. Rep.* **7**, 46018; doi: 10.1038/srep46018 (2017).

**Publisher's note:** Springer Nature remains neutral with regard to jurisdictional claims in published maps and institutional affiliations.

## Supplementary Material

Supplementary Information

## Figures and Tables

**Table 1:** Output parameters of the uniform design.

No.	Aqueous separation ratio (%)	Viscosity (mPa·s)	Suspensibility (%)	
			Before storage	After storage
U-1	3.92 ± 0.29 f	362.3 ± 1.8a	94.11 ± 1.59 ab	91.95 ± 1.76 a
U-2	6.27 ± 0.31e	300.0 ± 1.9c	92.89 ± 2.18 b	91.09 ± 2.53 a
U-3	7.92 ± 0.14d	278.1 ± 1.5e	94.34 ± 1.22 ab	93.10 ± 1.52 a
U-4	15.04 ± 0.24b	308.6 ± 1.5b	94.39 ± 1.30 ab	93.31 ± 1.19 a
U-5	2.75 ± 0.20 g	284.2 ± 2.7d	94.74 ± 2.16 ab	91.42 ± 2.11 a
U-6	6.47 ± 0.24e	359.6 ± 2.7a	95.16 ± 1.06 ab	92.92 ± 1.26 a
U-7	1.74 ± 0.24 h	364.9 ± 1.6a	97.62 ± 1.44 a	93.71 ± 6.36 a
U-8	30.63 ± 0.23a	267.8 ± 2.3 f	95.55 ± 1.61 ab	95.00 ± 2.71 a
U-9	13.60 ± 0.22c	302.3 ± 3.6c	96.40 ± 1.62 ab	94.19 ± 1.02 a

Note: Data are displayed as the mean ± SD (standard deviation). Data of the same index with different lowercase letters are significantly different at the *p* < 0.05 level based on Tukey’s test.

**Table 2:** Regression models of stepwise regression and partial least squares for the aqueous separation ratio.

Model	Regression equation	R^2^	F-value	Degree of freedom	*p*-value
Linear model of stepwise regression	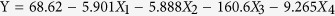	0.9571	22.33	4, 4	0.0054
Quadratic polynomial model of stepwise regression	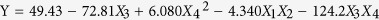	0.9810	51.57	4, 4	0.0011
Linear model of partial least squares	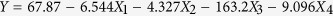	0.9401	—	—	—
Quadratic polynomial model of partial least squares	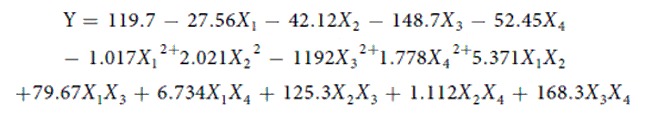	0.9595	—	—	—

Note: Non-significant factors were imported and eliminated at the *p* = 0.05 level. Y is the aqueous separation ratio and *X*_1_, *X*_2_, *X*_3_ and *X*_4_represent Tersperse2700, PE1601, xanthan gum and veegum, respectively. R^2^ represents the coefficient of determination.

**Table 3:** Verification tests.

No.	*X*_1_	*X*_2_	*X*_3_	*X*_4_	Aqueous separation ratio/%			Viscosity/mPa·s		
					Predicted value	Measured value	Relative error	Predicted value	Measured value	Relative error
1	2.50	0.50	0.21	0.70	13.44	8.17	64.50%	284.1	264.3	7.49%
2	2.50	0.70	0.22	1.10	3.12	3.45	9.57%	286.2	278.8	2.65%

Note: *X*_1_, *X*_2_, *X*_3_ and *X*_4_ represent the mass fractions of Tersperse2700, PE1601, xanthan gum and veegum, respectively.
