# Supplementary material for: Integrating uniform design and response surface methodology to optimize thiacloprid suspension
Source: Sci Rep. 2017 Apr 6;7:46018. doi: 10.1038/srep46018 (PMC5382544; doi:10.1038/srep46018)
Supplement: Supplementary Information [file srep46018-s1.doc]

Supplementary Information:

Integrating uniform design and response surface methodology to optimize thiacloprid suspension

Bei-xing Li,^1,2*^ Wei-chang Wang,^1,3*^ Xian-peng Zhang,^1,3^ Da-xia Zhang,^1,2^ Wei Mu,^2,3^ Feng Liu^1,3**^

1. Shandong Provincial Key Laboratory for Biology of Vegetable Diseases and Insect Pests, College of Plant Protection, Shandong Agricultural University, Tai’an, Shandong 271018, P. R. China

2. Research Center of Pesticide Environmental Toxicology, Shandong Agricultural University, Tai’an, Shandong 271018, China

3. Key Laboratory of Pesticide Toxicology & Application Technique, Shandong Agricultural University, Tai’an, Shandong 271018, P. R. China

^*^ B. Li and W. Wang share joint first authorship.

^**^ To whom correspondence should be addressed. Tel.: +86 0538-8242611.

E-mail address: fliu@sdau.edu.cn (F. Liu).


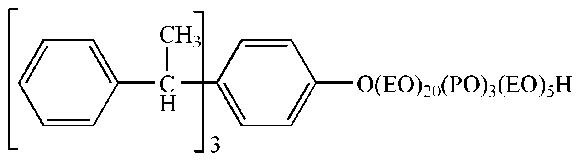


**Figure S1** Chemical structure of PE1601


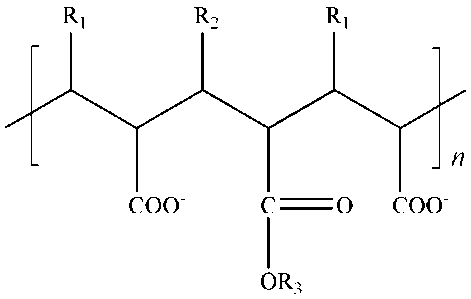


**Figure S2** Chemical structure of Tersperse2700 (R_1_, R_2_ and R_3_ are simple hydrophobic groups)


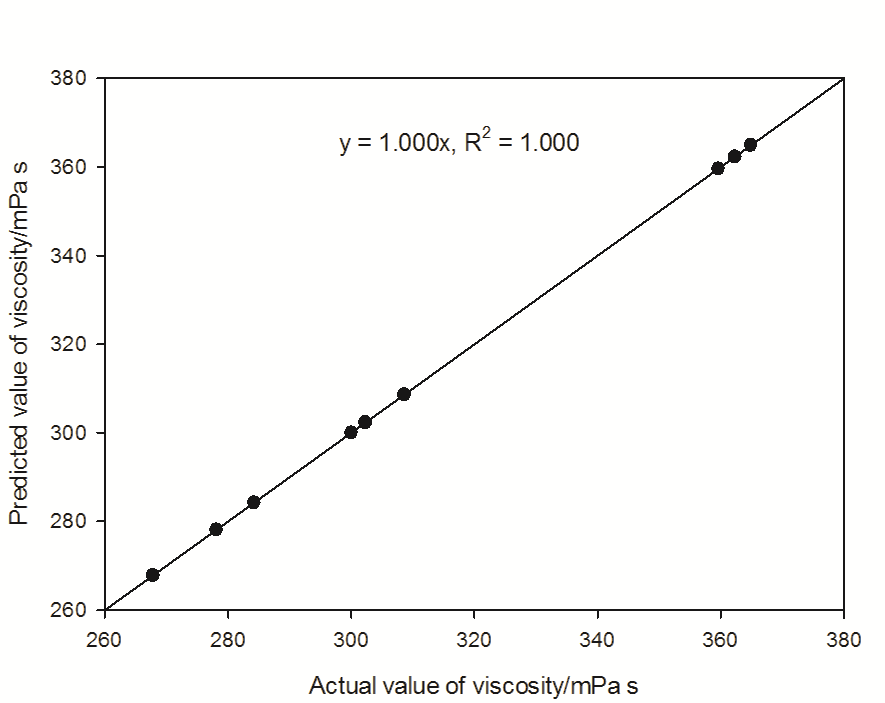


**Figure S3** Predicted and actual values for the viscosity

**Table S1** Size distribution of the tested samples

| No. | Before storage (μm) | | | After storage (μm) | | |
| --- | --- | --- | --- | --- | --- | --- |
|  | D_10_ | D_50_ | D_90_ | D_10_ | D_50_ | D_90_ |
| U-1 | 0.80 | 1.77 | 2.93 | 0.89 | 2.16 | 3.78 |
| U-2 | 0.71 | 2.47 | 5.45 | 0.81 | 2.96 | 6.74 |
| U-3 | 0.82 | 1.77 | 2.89 | 1.01 | 2.52 | 4.51 |
| U-4 | 0.83 | 1.88 | 3.16 | 0.90 | 2.05 | 3.46 |
| U-5 | 0.74 | 1.76 | 3.06 | 0.85 | 2.26 | 4.19 |
| U-6 | 0.83 | 1.88 | 3.17 | 0.84 | 2.02 | 3.54 |
| U-7 | 0.69 | 1.42 | 2.25 | 0.83 | 1.70 | 2.68 |
| U-8 | 0.77 | 1.62 | 2.6 | 0.82 | 1.74 | 2.83 |
| U-9 | 0.70 | 1.57 | 2.62 | 0.71 | 1.59 | 2.64 |

**Table S2** The regression models of stepwise regression and partial least squares for the viscosity

| Model | Regression equation | R^2^ | F-value | Degree of freedom | p-value |
| --- | --- | --- | --- | --- | --- |
| Linear model of stepwise regression | *Y* = 232.8 – 16.93*X*_1_+ 33.80*X*_2_ + 104.6*X*_3_ + 45.74*X*_4_ | 0.9977 | 430.7 | 4, 4 | 0.0001 |
| Quadratic polynomial model of stepwise regression | *Y* = 244.5 + 17.94*X*_3_ + 51.57*X*_4_ - 4.100*X*_1_^2^ + 2.032*X*_2_^2^ + 122.3*X*_2_*X*_3_ + 5.113*X*_2_*X*_4_ - 70.87*X*_3_*X*_4_ | 1.000 | 7.143×10^4^ | 7, 1 | 0.0029 |
| Linear model of partial least squares | *Y* = 271.2 - 20.98*X*_1_ + 30.70*X*_2_ - 30.85*X*_3_ + 44.50*X*_4_ | 0.9807 | - | - | - |
| Quadratic polynomial model of partial least squares | *Y* = - 233.7 + 180.4*X*_1_ + 113.8*X*_2_ + 2452*X*_3_ + 98.76*X*_4_ - 3.986*X*_1_^2^ - 8.858*X*_2_^2^ + 555.3*X*_3_^2^ + 13.40*X*_4_^2^ - 1.993*X*_1_*X*_2_ - 844.8*X*_1_*X*_3_ - 20.59*X*_1_*X*_4_ - 399.7*X*_2_*X*_3_ + 4.988*X*_2_*X*_4_ - 378.9*X*_3_*X*_4_ | 0.9606 | - | - | - |

Note: Importing and eliminating non-significant factors were set at *p* = 0.05 level. *Y* is aqueous separation ratio, *X*_1_, *X*_2_, *X*_3_ and *X*_4_ represent Tersperse2700, PE1601, xanthan gum and veegum, respectively. R^2^ represents the coefficient of determination.

**Table S3** Output and total desirability

| No. | Individual desirability of aqueous separation ratio | Individual desirability of viscosity | Total desirability |
| --- | --- | --- | --- |
| U-1 | 0.9245 | 0.0268 | 0.1573 |
| U-2 | 0.8432 | 0.6684 | 0.7507 |
| U-3 | 0.7861 | 0.8939 | 0.8383 |
| U-4 | 0.5396 | 0.5798 | 0.5594 |
| U-5 | 0.9650 | 0.8311 | 0.8956 |
| U-6 | 0.8363 | 0.0546 | 0.2137 |
| U-7 | 1 | 0 | 0.0000 |
| U-8 | 0 | 1 | 0.0000 |
| U-9 | 0.5895 | 0.6447 | 0.6165 |

**Table S4** The regression models of stepwise regression and partial least squares for overall desirability

| Model | Regression equation | R^2^ | F-value | Degree of freedom | *p*-value | Residual |
| --- | --- | --- | --- | --- | --- | --- |
| Linear model of stepwise regression | *Y* = -0.2420 + 0.3450*X*_1_ | 0.4350 | 5.390 | 1, 7 | 0.0533 | - |
| Quadratic polynomial model of stepwise regression | *Y* = -0.01650 + 1.736*X*_1_*X*_3_ - 0.1122*X*_2_*X*_4_ | 0.7595 | 9.472 | 2, 6 | 0.0139 | - |
| Linear model of partial least squares | *Y* = -0.7918 + 0.3265*X*_1_ - 0.09799*X*_2_ + 4.884*X*_3_ - 0.1453*X*_4_ | 0.6777 | - | - | - | 6.576 |
| Quadratic polynomial model of partial least squares | *Y* = 0.03256 + 0.1003*X*_1_ + 0.9996*X*_2_ - 15.21*X*_3_ + 1.268*X*_4_ - 0.01874*X*_1_^2^ - 0.006375*X*_2_^2^ + 36.96*X*_3_^2^ - 0.1634*X*_4_^2^ - 0.2819*X*_1_*X*_2_ + 4.222*X*_1_*X*_3_ - 0.1900*X*_1_*X*_4_ - 1.439*X*_2_*X*_3_ - 0.2095*X*_2_*X*_4_ - 1.801*X*_3_*X*_4_ | 0.9219 | - | - | - | 6.632 |

Note: Importing and eliminating non-significant factors were set at *p* = 0.05 level. *Y* is overall desirability, *X*_1_, *X*_2_, *X*_3_ and *X*_4_ represent Tersperse2700, PE1601, xanthan gum and veegum, respectively. R^2^ represents the coefficient of determination.

**Table S5** Factors and levels of the uniform design

| No. | Tersperse2700 | | PE1601 | | Xanthan gum | | Veegum | |
| --- | --- | --- | --- | --- | --- | --- | --- | --- |
|  | Level | Amount/% | Level | Amount/% | Level | Amount/% | Level | Amount/% |
| U-1 | 1 | 1.00 | 7 | 2.00 | 8 | 0.21 | 6 | 1.25 |
| U-2 | 6 | 2.25 | 1 | 0.50 | 7 | 0.20 | 7 | 1.50 |
| U-3 | 9 | 3.00 | 3 | 1.00 | 4 | 0.17 | 5 | 1.00 |
| U-4 | 4 | 1.75 | 6 | 1.75 | 1 | 0.14 | 4 | 0.75 |
| U-5 | 8 | 2.75 | 5 | 1.50 | 9 | 0.22 | 3 | 0.50 |
| U-6 | 3 | 1.50 | 4 | 1.25 | 5 | 0.18 | 9 | 2.00 |
| U-7 | 7 | 2.50 | 8 | 2.25 | 2 | 0.15 | 8 | 1.75 |
| U-8 | 2 | 1.25 | 2 | 0.75 | 3 | 0.16 | 2 | 0.25 |
| U-9 | 5 | 2.00 | 9 | 2.50 | 6 | 0.19 | 1 | 0 |
